# COVID-19 control pitfalls and challenges and drivers of SARS-CoV-2 transmission in Zimbabwe

**DOI:** 10.11604/pamj.2021.38.28.25758

**Published:** 2021-01-13

**Authors:** Grant Murewanhema

**Affiliations:** 1 Department of Obstetrics and Gynaecology, College of Health Sciences, University of Zimbabwe, Harare, Zimbabwe

**Keywords:** SARS-CoV-2, COVID-19, Zimbabwe

## Abstract

Despite numerous public health interventions introduced by the Zimbabwean government, the COVID-19 burden continues to grow. The number of confirmed cases increased from less than 600 to over 6000 in a period of two months, and the fatalities now exceed 150. The source of infection has significantly changed from imported cases to community transmission. The greatest burden of COVID-19 is in the country’s two biggest provinces, Harare and Bulawayo, and differentiated approaches are urgently required to curb further transmissions whilst allowing other aspects of the population’s livelihood to continue. We discuss some of the pitfalls and challenges for COVID-19 control, and the possible drivers of SARS-CoV-2 community transmission in the country. An urgent multi-sectoral intersection to effectively deal with these caveats is required, and political commitment to deal with the crisis remains an indispensable variable.

## Perspective

Severe acute respiratory syndrome coronavirus 2 (SARS-CoV-2) is the aetiological virus for coronavirus disease 2019, popularly known as COVID-19 [1]. From the initial cases detected in Wuhan in December 2019, the outbreak of this novel virus rapidly evolved to become a global pandemic by March 2010 [2,3]. World Health Organization (WHO) situation reports indicate that by 24 August 2020, an estimated 21 million individuals had contracted SARS-CoV-2, with slightly over 800 000 fatalities [4]. Another estimated 1.7 million individuals had contracted the virus in the preceding 7 days [4], making SARS-CoV-2 the most aggressive virus ever, with a high effective reproduction number R_0_. Africa is the fifth most affected continent, with slightly over one million cases, constituting an estimated four percent of the global burden, with about 20 000 fatalities [4]. Zimbabwe reported the first cases of COVID-19 in March 2020, which were all imported [5]. The trajectory was very slow in the first 3-4 months of the pandemic, with less than 600 cases by the end of June 2020. Despite scholarly projections that Zimbabwe would reach 1000 cases by mid-April 2020 [6]. The confirmed cases were still less than 60% of the projection. The majority of cases during this period were imported, as indicated by the daily situation reports from the Ministry of Health and Child Care (MOHCC), and the highest number of returning residents were from the neighboring South Africa. However, since July 2020, the number of reported cases increased significantly, with over 100 cases confirmed per day in many instances. The balance of infection source shifted heavily towards locally acquired infections as Zimbabwe entered into the dreaded phase of community SARS-CoV-2 transmission. As of 25 August 2020, the cumulative number of cases in Zimbabwe stood at 6196, with 166 fatalities, and a case fatality ratio of 2.7% and 80% recovery rate according to the WHO de-isolation criteria [7]. Harare Province contributed an estimated 40% of the disease burden, and 56% of the fatalities, and Bulawayo Province was the second most affected with 20% of the cases and 17.5% of the fatalities. The other eight provinces contributed 40% of the infection burden, and 62.5% of the deaths. Controlling transmission effectively requires an urgent evaluation and understanding of the infection dynamics, including hotspots and possible drivers for new infections. As the country is now in community transmission, differentiated models of control are required based on local transmission patterns. Unfortunately, no formal research has been published to date to explore this situation, and the focus has been on quarantining returning travelers, and testing, treating and isolating confirmed cases. We describe the mitigating public health interventions introduced by the Zimbabwean government since March 2020, and discuss the pitfalls and challenges for control, and the possible drivers of transmission since the beginning of the outbreak in Zimbabwe.

**Responses to the COVID-19 pandemic by the Zimbabwean government**: a state of national disaster was pronounced at the outset of the pandemic, culminating in a total lockdown effected on 30 March 2020 [8]. The initial 21-day lockdown period meant stoppage of all non-essential services except for healthcare and security [5]. Local and international travel was banned, with the requirement for exemption letters at road checks. Retail outlets were permitted to operate for restricted hours during the day with screening and sanitizing of hands at entrances. The sale of alcoholic beverages was banned. The lockdowns have been gradually relaxed, with resumption of most services under strict regulations. Mass gatherings, including religious gatherings were initially banned but are now permitted with not more than 50 congregants. On 04 April 2020, mandatory wearing of facemasks in all public places was pronounced. Returning residents were mandated to go into quarantine at designated facilities throughout the country [5]. Rapid response teams were activated to respond swiftly to rumors and alerts, and surveillance teams were constituted to facilitate contact tracing, follow-up, and case investigation. An expanded testing strategy was released in April 2020 to increase the scope of surveillance, and detect and isolate confirmed cases, and protect healthcare workers and patients [9]. Despite a comprehensive testing strategy, implementation has been complicated by regular stock outs of essential testing reagents. Targets to test 40 000 people per month as was proposed in the Zimbabwe COVID-19 operational plan [5] have not been met. Zimbabwe imports test kits for COVID-19 and provincial testing capacitation through GeneXpert platforms has not materialized adequately. The requirement for mandatory quarantine of returning residents has been removed as the need to deal with community transmission has grown.

### Pitfalls, challenges and drivers of SARS-CoV-2 transmission

**Quarantine centers:** the primary purpose of the quarantine centers was to house returning residents for the duration of the incubation period, and contain any possible infections in these centers to avoid local transmission. However, the conditions in the quarantine centers were anecdotally reported as unsuitable [10]. Reports of overcrowding, sharing of amenities and sexual activities within the quarantine centers were noted. There were multiple reports on inmates running away from inhumane conditions in the centers and escaping into communities. Administrative factors including mixing of different cohorts and delayed release of results further complicated the situation, as inmates went beyond 21 days without results. During the early phases of the outbreak in Zimbabwe, the highest number of cases were diagnosed in quarantine centers; therefore, it is possible they were the sources of infection.

**Porous borders:** Zimbabwe shares borders with a number of neighbors, including South Africa, Botswana, Zambia, Malawi and Mozambique. Though the actual numbers are unknown, a number of residents entered illegally into their communities through porous borders and escaped mandatory quarantine. Some who were reported by their communities subsequently tested positive for SARS-CoV-2, and possibly may have been contagion sources for the community.

**Testing:** the testing capacity falls grossly short of expectations. Real-time polymerase chain reaction (RT-PCR) testing is scarce and expensive, and the role of antibody testing in the management of COVID-19 remains controversial [11], with the world health organization advising rapid detection tests (RDTs) to be reserved for academic research and community surveillance [12]. RDTs can have a high incidence of false negatives who subsequently turn positive on RT-PCR. The testing in the country remains centralized to reference laboratories in Harare and Bulawayo. Shortage of cartridges has hampered efforts to capacitate GeneXpert testing at provincial level. Therefore, the reference labs remain overwhelmed with backlogs. Sometimes, patients have to go beyond ten days without receiving their results. Meanwhile, especially considering that the initial situation reports indicated that most cases were asymptomatic, they could pass on SARS-CoV-2 unknowingly.

**Population dynamics:** Africa has a relatively young population [13]. It is postulated that young people who contract SARS-CoV-2 may remain asymptomatic for COVID-19 and be drivers of transmission [13]. In the initial phases, the majority of cases were confirmed among young people returning from other countries for economic reasons and they were reported as asymptomatic, and deaths in the young are rare. With the thriving informal sector driven by young people in Zimbabwe, silent SARS-CoV-2 infections maybe a common occurrence.

**Public health sector challenges:** the public health sector in Zimbabwe faces many challenges. Constant industrial actions by healthcare personnel, shortages of equipment, medicines and sundries, including personal protective equipment (PPE) are widely reported [14]. These may have been further worsened by supply chain disruptions owing to travel restrictions [15]. Prices of imported commodities including medicines may go up by 10-25%. Fear and anxiety by healthcare practitioners further worsens the situation [16]. Public health facilities have not been offering clinical services regarded as non-emergency, and patients without COVID-19 test results have been denied services, the majority of whom are not on medical insurance and cannot afford private testing. People have been forced to look after their sick relatives at home without adequate infection prevention control measures, which could potentially result in widespread community transmissions. Without adequately dealing with healthcare issues, COVID-19 control is likely to remain a challenge.

**Risk perception:** an unpublished report by the risk communication and community engagement (RCCE) pillar observed low risk perception among Zimbabweans, especially the young. People believe what they see, and similar to the HIV epidemic, people would not believe it was a reality until they lost close family members. Similarly, there are some who still believe that COVID-19 is not an African disease. Low risk perception serves as an inhibitor fo

r the uptake of preventive measures and must be addressed urgently. Physical distancing and wearing of masks in public places such as supermarkets and banks remains a huge challenge.

**Economic and social challenges:** the majority of the working population depends on public transport to go to work. Most forms of public transport were banned at the beginning of the lockdown period, except for those affiliated to the Zimbabwe united passenger company, which unfortunately does not have enough vehicles to service the public. Overcrowding in these buses is common and does not permit physical distancing. A good proportion of the population in the high-density suburbs of Harare and Chitungwiza fetches water from community boreholes where people queue for long hours without physical distancing and facemasks. With an unemployment rate estimated to be in excess of 90%, and with the hyperinflationary economic environment, many have taken to the informal sector for livelihood. There is no basic infection screening including temperature checks; there are no facilities for hand hygiene, common ablution facilities shared are not hygienic, and physical distancing is a luxury. High unemployment rate means many cannot afford to seek treatment on time, and may drive SARS-CoV-2 community transmission before access to testing and results.

**Political challenges:** political commitment to dealing with a national security threat entails prioritisation of resource allocation to the health sector to effectively deal with the ongoing challenges. It also entails wide consultative forums with public health experts to obtain evidence-based recommendations to effectively deal with the pandemic. Whilst political commitment remains an indispensable variable of the COVID-19 pandemic control, the scientific world must be allowed to take the lead.

**Surveillance and operational research:** generation of local evidence is a critical missing link between SARS-CoV-2 community transmission and control. Documenting challenges and pitfalls, strengths and best practices, and meaningful analysis of available data is the crux of evidence-based public health and clinical practice for COVID-19. The academia must be regarded as an important sector to advice the research pillar, and this must be prioritized as differentiated interventions are urgently required to deal with the pandemic whilst allowing other aspects of people´s livelihoods to continue.

## Conclusion

Dealing with the COVID-19 pandemic and curbing further spread of SARS-CoV-2 remains an urgent need in Zimbabwe. Controlling community transmission requires differentiated approaches premised on comprehension of the pitfalls and challenges for control, and the drivers for transmission. An urgent discourse between politics, science, academia and public health is needed for the generation and implementation of evidence-based public health interventions to effectively deal with the pandemic.
